# Translational Evaluation of a Machine Learning-Based Interactive Lab for Aphasia Rehabilitation in Post Stroke Patients

**DOI:** 10.1109/JTEHM.2025.3638643

**Published:** 2025-11-28

**Authors:** Mukul Kumar, Rei-Zhe Wu, Shih-Ching Yeh, Eric Hsiao-Kuang Wu, Po-Yi Tsai

**Affiliations:** Department of Computer Science and Information EngineeringNational Central University 34911 Taoyuan 320317 Taiwan; Department of Physical Medicine and RehabilitationTaipei Veterans General Hospital Taipei 112201 Taiwan

**Keywords:** Aphasia, stroke rehabilitation, translational engineering, machine learning, digital health, interactive therapy, health informatics

## Abstract

Objective: To address the limitations of conventional aphasia therapy by developing and clinically evaluating a machine learning based interactive lab for personalized rehabilitation in post-stroke patients. Methods and Procedures: A four week clinical trial was conducted with 27 aphasia patients, randomly assigned to an experimental group ( $n=11$) using the Language Interactive Lab and a control group ( $n=16$) receiving conventional therapy. Language performance was assessed using the Chinese Communicative Aphasia Test (CCAT). System interaction data were also used to train classifiers for aphasia severity and recovery tracking. Results: The experimental group showed statistically significant improvements in 7 out of 9 CCAT subtests ( $p < 0.05$) and a highly significant total score increase ( $p < 0.001$) compared to the control group. Machine learning classifiers achieved up to 91.7% accuracy in predicting aphasia severity and recovery progression. Conclusion: The proposed interactive lab integrates gamified therapy with real time, explainable machine learning assessment, demonstrates clinical efficacy in improving language outcomes, and offers a scalable framework for AI-driven, adaptive neurorehabilitation that has been clinically validated within a hospital setting and designed to align with Taiwan Food and Drug Administration (TFDA) software-as-a-medical-device (SaMD) regulatory principles for translational deployment in clinical environments and hospital investigational use guidelines. Clinical Impact—The integration of gamified digital therapy with machine learning analytics supports personalized, data driven intervention for aphasia rehabilitation in both clinical and home settings, particularly in resource limited environments. Clinical and Translational Impact Statement—This study supports Clinical Research by demonstrating that AI-powered digital therapy significantly improves language outcomes in post-stroke aphasia patients and offers a pathway to scalable, at home neurorehabilitation.

## I. Introduction

Aphasia is a disabling neurogenic language disorder, typically caused by stroke, affecting 20–25% of stroke survivors worldwide [1]. It impairs core communication functions speaking, listening, reading, and writing due to damage in brain regions responsible for language processing. Clinically, patients may show reduced fluency, word retrieval difficulties, comprehension deficits, phonemic errors, and semantic paraphasias [2]. Aphasia is classified into types such as Broca’s, Wernicke’s, global, and transcortical aphasia, based on fluency, comprehension, and repetition abilities [3], each linked to specific lesion sites in the left hemisphere. Fig. I, illustrates this classification using a decision tree that maps aphasia subtypes based on fluency, comprehension, and repetition, aiding in clinical differentiation based on observable language features.

**FIGURE 1.**
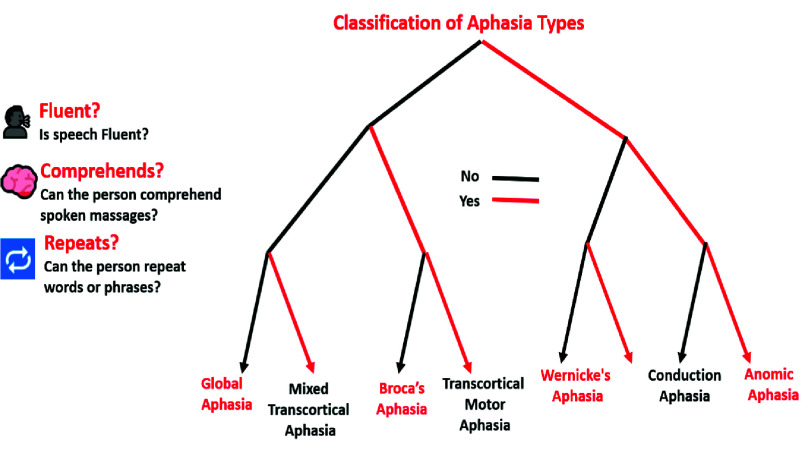
Decision tree for classification of aphasia subtypes based on speech fluency, auditory comprehension, and repetition ability. This structure aids in differentiating among major aphasia types using observable language features.

Aphasia’s long term effects go beyond language deficits, often causing reduced independence, social isolation, and increased caregiver and healthcare burden. Conventional therapy relies on clinician administered interventions and subjective assessments, which are resource intensive, inconsistently applied, and often inaccessible in remote or underserved areas [4], [5], [6]. These limitations drive the need for scalable, objective rehabilitation tools that deliver effective therapy with real time, measurable monitoring. In this context, computer assisted platforms augmented with ML show promise for outcome prediction and progress tracking. This study introduces the Language Interactive Lab and evaluates its clinical impact and predictive capabilities using behavioral and linguistic metrics.

Human language processing is primarily localized in the left hemisphere, which governs core linguistic functions, while the right hemisphere supports nonverbal and higher order cognition [3]. Consequently, left hemisphere lesions commonly due to ischemic or hemorrhagic strokes are the primary cause of aphasia. Key language areas include Broca’s area (left inferior frontal gyrus near the Sylvian fissure), responsible for production and syntax, and Wernicke’s area (posterior left superior temporal gyrus), which governs comprehension and grammar [7]. Aphasia diagnosis and treatment require a multidisciplinary approach. Clinical evaluations assess auditory comprehension, expression, reading, writing, and related motor cognitive skills. Impairment type and severity guide personalized rehabilitation strategies. Traditional therapy includes structured exercises, verbal cueing, and task based drills. More recently, neuromodulatory techniques like transcranial magnetic stimulation (TMS) and transcranial direct current stimulation (tDCS) have been explored [8], [9], aiming to promote neuroplasticity and facilitate reorganization of language networks to enhance recovery beyond behavioral therapy alone.

Aphasia affects more than communication it deeply impacts emotional well being, social participation, and overall quality of life [2]. It places long term psychological, social, and physical burdens on both patients and caregivers, often leading to emotional distress, identity loss, and social isolation [10], [11]. Modern neurorehabilitation emphasizes these psychosocial aspects, promoting personalized, patient centered care through compensatory strategies, group therapy, assistive technologies, and caregiver training. Despite the vital role of speech language pathologists (SLPs), clinician shortages, high caseloads, and reliance on time consuming, paper based tools like the CCAT limit access to consistent, high quality care especially in under resourced or mobility restricted settings.

To address these barriers, digital health interventions offer scalable alternatives to traditional therapy. Computer assisted and virtual reality (VR) based platforms have shown promise in improving engagement, motivation, and outcomes in neurological conditions like ADHD, concussion, and post stroke rehabilitation [12], [13]. Meanwhile, advances in big data and AI have enabled machine learning to uncover patterns in behavioral and linguistic data for accurate classification, progress tracking, and outcome prediction [14]. Wearable sensors further enhance these systems by providing real time, fine grained feedback, boosting the clinical utility and reliability of AI assisted neurorehabilitation [15].

This study presents and clinically evaluates the Language Interactive Lab, a computer based aphasia therapy platform featuring four gamified modules, semantic comprehension, associative matching, auditory vocabulary recognition, and lexical expression. These modules target core language deficits while capturing rich behavioral data during use. By integrating task performance with machine learning classifiers, the system enables real time, objective progress tracking and supports personalized, data driven therapy. A four week randomized clinical trial was conducted with post stroke aphasia patients, comparing an experimental group using the system to a control group receiving conventional therapist led care. Collected behavioral data were used to train interpretable ML models for classifying aphasia severity and monitoring recovery, enhancing the platform’s diagnostic and predictive utility.

The primary contributions of this work are threefold,

1) The design of four gamified, ML compatible language rehabilitation modules targeting key deficits.
2) Development of explainable ML models for aphasia severity classification using in task behavioral data.
3) Clinical validation demonstrating both therapeutic effectiveness and diagnostic utility.

In addition, the Language Interactive Lab represents a translational engineering effort that bridges algorithmic innovation with real world clinical deployment. It integrates gamified therapy modules with real time, explainable machine learning (XAI) assessment, enabling clinicians to interpret how model decisions correspond to linguistic and behavioral patterns. The system has been hospital validated as a SaMD prototype, developed in alignment with Taiwan Food and Drug Administration (TFDA) regulatory expectations and international standards (IEC 62304 for software life-cycle processes and ISO 14971 for risk management). This compliance framework ensures clinical safety, data traceability, and cybersecurity, positioning the platform for scalable deployment across both clinical and home rehabilitation settings, where continuous monitoring and personalized, adaptive feedback can support long-term recovery.

The remainder of this paper is organized as follows: Section II reviews related work, Section III describes the proposed method and system design, Section IV presents the experimental results, and Section V concludes with future directions.

## II. Related Works

### A. Aphasia Therapy and Clinical Practices

Aphasia is a common post stroke impairment, with severity ranging from mild semantic deficits to severe expressive challenges depending on lesion location [16]. Mild cases benefit from synonym drills and vocabulary tasks, while severe cases require phonological and writing support. Combining high and low intensity therapy engages both automatic and effortful language pathways, improving outcomes [17], [18]. However, conventional therapy depends heavily on SLPs, making it labor intensive and difficult to scale particularly in under resourced settings. This has led to a shift toward accessible, tech driven interventions that maintain clinical efficacy while reducing therapist burden.

### B. Digital Tools for Aphasia Rehabilitation

Digital platforms provide scalable, adaptive alternatives to therapist led interventions. One UK developed software offers 10,000 + semantic, auditory, and visual exercises, improving image naming among stroke survivors [19], [20]. However, broader language domains like comprehension remain under assessed. To address this, the Tavistock Trust’s Aphasia Software Finder curates validated tools, including an iPad based program shown to improve CAT scores and spontaneous speech [21], [22]. Neuromodulatory approaches like repetitive transcranial magnetic stimulation (rTMS) have also shown promise. rTMS modulates cortical excitability and supports reorganization of language networks [23], [24], [25], with sustained gains reported in naming and semantic comprehension [26]. Still, rTMS remains costly and equipment-intensive, limiting accessibility.

### C. Machine Learning and Explainable AI in Aphasia Assessment

Though rTMS and digital tools show promise, they often focus on isolated functions or require clinician oversight. Traditional assessments also rely on expert interpretation, introducing subjectivity. ML offers a scalable alternative by analyzing behavioral and linguistic data for real time classification and recovery prediction [14], [27], [28]. However, few studies explore task level performance or integrate ML into live therapy. Existing models often lack interpretability, hindering clinical use. To bridge this gap, we propose an automated framework combining gamified therapy modules with ML analytics. Four interactive modules target core language functions, and interaction data are used to train interpretable models for severity estimation and recovery tracking. To enhance transparency, we incorporate Explainable AI techniques that highlight features influencing predictions [29], supporting clinician validation and aligning model output with clinical reasoning.

## III. Methodology

**FIGURE 2.**
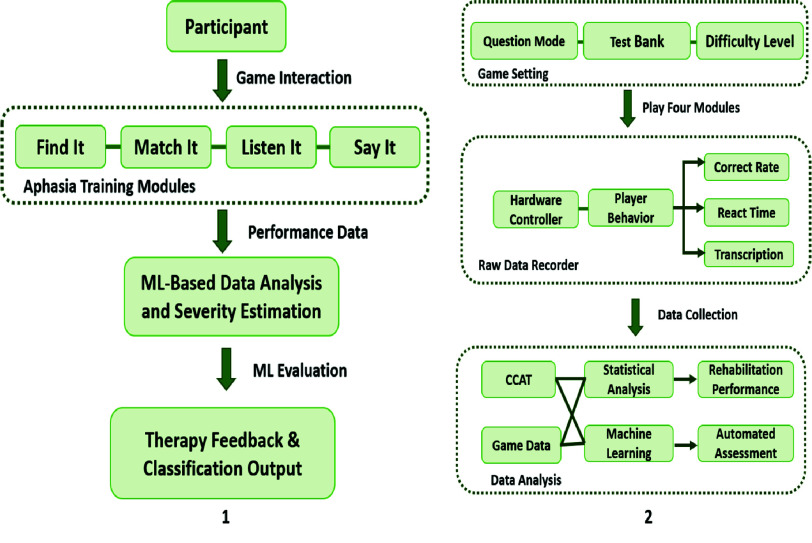
End-to-end architecture of the language interactive lab for aphasia rehabilitation. The diagram illustrates client-side interactions (module engagement and audio capture), backend data processing (logging, feature extraction), machine learning analysis (model training and inference), and clinician-facing output interpretation with explainability overlays.

The aphasia rehabilitation system comprises three main stages, module configuration, data acquisition, and performance analysis, as shown in Fig. 2, which illustrates the backend processing and ML based evaluation pipeline. Four interactive rehabilitation modules were developed, and each patient completed all modules. Behavioral data collected in real time during sessions were used to train and update ML models for severity classification and progress tracking, with explainability overlays enabling therapists to interpret feature contributions behind each prediction. A detailed end-to-end system architecture diagram Fig. 2, summarizes the complete data flow from patient interaction on the client side to backend processing, feature extraction, model training, and clinician-facing interpretation.

### A. Module Configuration

Clinical consultations at Taipei Veterans General Hospital emphasized the value of module diversity in sustaining patient engagement. Each training module includes configurable question banks and customizable visual styles from cartoon like to photorealistic that therapists can tailor to individual preferences. The system’s Battery of Tests function allows therapists to personalize training by selecting question sets from semantic categories like fruits, animals, and daily objects. Difficulty levels are adjustable to align with each patient’s language proficiency in comprehension, expression, reading, and writing.

### B. Data Acquisition

The four interactive therapy modules were developed using Unity Engine (v2020.3.33) and support Windows based systems. Optimized for low hardware requirements, the platform runs on standard personal computers for both clinical and home use. Patients interact via mouse, touchscreen, or tablet, with a microphone used to capture verbal responses during expression tasks. Each session automatically logs detailed performance metrics, including per question accuracy and reaction time. Audio responses from naming tasks are also stored for later acoustic and linguistic analysis to support ML based classification.

### C. Module Scenarios and Task Design

**FIGURE 3.**
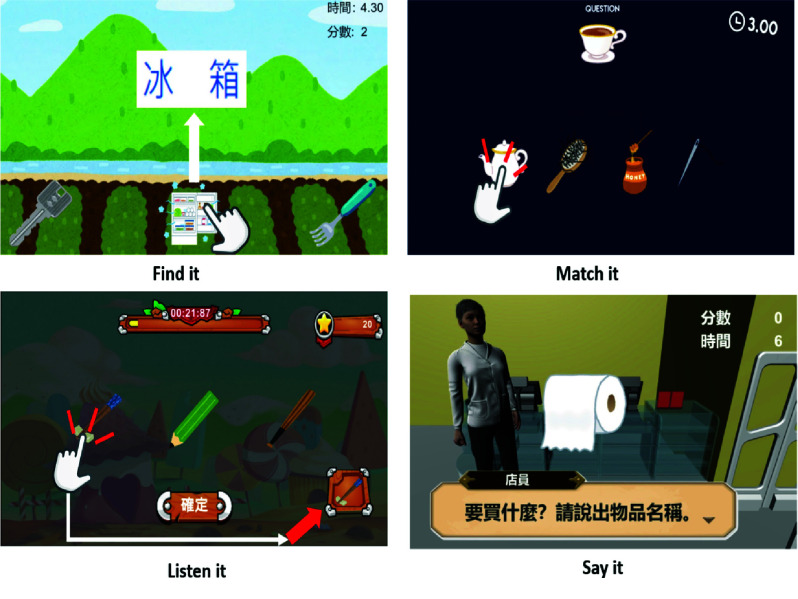
Interactive interfaces of the find it, match it, listen it, and say it modules for multimodal language rehabilitation.

1) **Find It:** This semantic comprehension module Fig. 3, presents a text prompt, requiring the patient to drag the corresponding image to a response area. It targets skills such as semantic association, symbol object matching, reading comprehension, and verbal repetition.
2) **Match It:** This associative semantic matching module Fig. 3, presents a text prompt and requires patients to select one or more matching images. It reinforces descriptive labeling, semantic association, and verbal repetition.
3) **Listen It:** This vocabulary comprehension module Fig. 3, uses audio prompts to focus on item identification and quantity recognition. Patients respond by selecting and adjusting images. It targets listening comprehension, semantic matching, descriptive labeling, and verbal repetition.
4) **Say It:** This lexical expression module Fig. 3, presents prompts via audio and text, requiring verbal responses evaluated by a built in speech recognition engine. Simulating a supermarket scenario, patients name items, specify quantities, and calculate total cost using basic multiplication. If answers are incorrect, the system provides sequential hints descriptive cues, initial letters, and lip reading videos. This multimodal feedback supports expressive and receptive language skills, targeting functions such as labeling, semantic matching, listening, oral expression, repetition, and reading comprehension.

### D. Experiment Design

**FIGURE 4.**
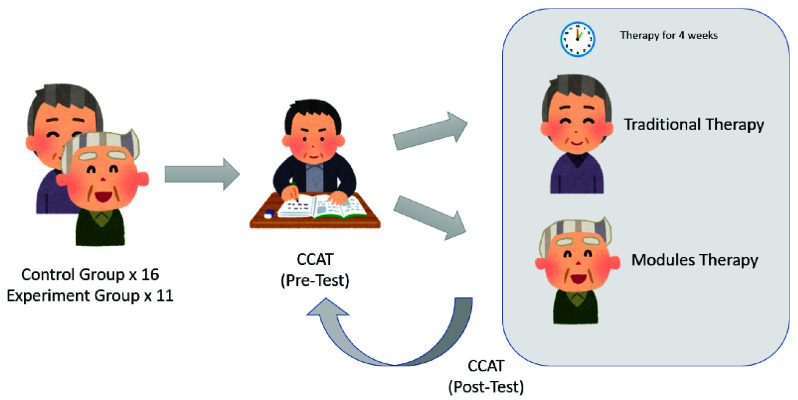
Experimental procedure and participant flow.

1) **Participants:** The study included 27 aphasia patients, divided into a control group ( $n = 16$) receiving conventional therapist led training, and an experimental group ( $n = 11$), which comprised eight aphasia only cases and three with co-occurring dysarthria Table 1. Demographic and clinical details are summarized in Table 2. The groups were comparable in sex, age, post stroke duration, stroke type, aphasia classification, and baseline CCAT scores, with no significant differences observed ( $p > 0.05$).
2) **Procedure:** As shown in Fig. 4, all participants completed the CCAT assessment before starting therapy. Baseline comparisons showed no significant difference in pre intervention CCAT scores between groups. Both groups then underwent a four week therapy program with 14 sessions. The control group received standard therapist led care, while the experimental group used the four interactive modules developed in this study. Therapists provided a brief orientation on module usage to ensure patient comprehension. After the program, all participants were reassessed using the CCAT to compare language recovery outcomes.
3) **CCAT Scale:** The CCAT is a standardized, culturally validated tool for assessing Mandarin speaking aphasia patients. It evaluates four core domains listening, speaking, reading, and writing across nine subtests, Conservation (auditory comprehension), Description (language clarity), Matching (semantic association), Listening Comprehension, Expression (oral output), Reading Comprehension (action based word recognition), Repetition, Imitation Writing (motor linguistic skills), and Spontaneous Writing. Each subtest is scored from 0 to 12, and total scores categorize aphasia severity into seven levels, slight deficit (12), mild (10–11), mildly moderate (9–10), moderate (7–9), moderately severe (5–7), severe (3–5), and extremely severe (0–3). The CCAT is widely used in clinical research to assess therapy effectiveness and monitor progression.

**TABLE 1:** Participant Information

Participant Information	Gender	Average ages	Count
Control Group	Male	60.54	11
	Female	69.6	5
Experiment Group	Male	63.27	9
	Female	72.75	2

**TABLE 2:** Demographic Data and Clinical Variables of Participants

Characteristic	All (N=27)	Control (n=16)	Experiment (n=11)	p-value
Male sex, n (%)	20 (74%)	11 (69%)	9 (82%)	0.79
Age (years), mean (SD)	65.1 (9.5)	64.6 (11.0)	65.8 (7.9)	0.722
Post-stroke duration (months), mean (SD)	19.6 (24.6)	23.5 (31.7)	13.8 (14.1)	0.28
Stroke type				
Infarction / Hemorrhage / Mixed	18 / 5 / 4	11 / 2 / 3	7 / 3 / 1	0.529
Aphasia type				
Broca	14	7	7	–
Transcortical motor	1	1	0	–
Wernicke	1	1	0	–
Global	4	3	1	–
Transcortical mixed	5	4	1	–
Dysphasia	2	0	2	0.778
Pre-CCAT total score, mean (SD)	64.4 (20.7)	60.7 (25.2)	69.7 (14.1)	0.235

**FIGURE 5.**
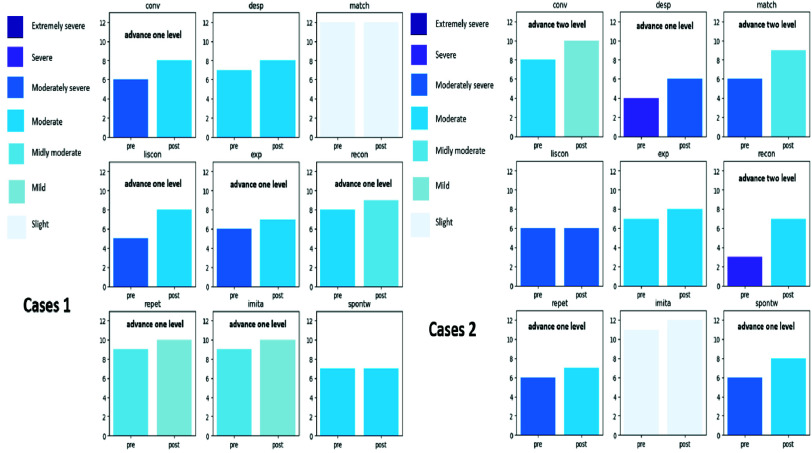
Patient representatives with significant progress (Cases 1 and 2).

### E. Data Analysis

1) **Feature Extraction:** After each module, patient performance data GameData, were automatically recorded at the question level, capturing task duration, reaction time, and response accuracy. For machine learning analysis, four statistical features were extracted, overall mean reaction time, mean correct reaction time, mean incorrect reaction time, and correct rate (proportion of correct responses), as defined in Table 3. Language functions targeted by each module are detailed in Table 4, based on input from clinical experts. Specifically, Find It supports semantic comprehension through text-to-image matching. Match It reinforces object association. Listen It targets auditory comprehension via action-based responses, and Say It trains oral expression and sentence construction. Additionally, task duration and error patterns offer insights into problem-solving ability, operational efficiency, and completion speed.
2) **Machine Learning:** Two classification pipelines were developed, one using CCAT subtest scores and another using behavioral performance data (GameData) from the therapy modules (Find It, Match It, and Listen It). Each model classified participants into aphasia or dysarthria groups. Classifiers including k-NN, SVM, RF, LR, and NN were implemented using Python’s scikit-learn library. To reduce overfitting, 10-fold cross-validation was applied and repeated 10 times. Among all models, Logistic Regression achieved the highest accuracy (91.7%) with CCAT subtest scores, while k-NN performed best using in game behavioral features (up to 74.1%). The lower accuracy of the GameData based models compared with CCAT based models (74.1 % vs 91.7%) reflects the greater behavioral variability and contextual noise of spontaneous gameplay. In game metrics capture unconstrained cognitive and motor responses such as hesitation, fatigue, or device familiarity that introduce variance absent in standardized CCAT tests. This observation suggests the need for refined feature design to stabilize behavioral embeddings and enhance diagnostic reliability. To ensure explainability, we applied model specific coefficients LR and model agnostic techniques (permutation importance and SHAP-style local attributions) to quantify per feature contributions (correct rate, reaction time profiles) for each decision. These XAI summaries were mapped to clinical constructs (auditory comprehension, naming, semantic association), allowing therapists to verify that model evidence aligns with language function deficits observed during sessions.

**TABLE 3:** GameData Feature

GameData Feature	Description
MT	Reaction time for each question
MCT	Reaction time for Correct question
MWT	Reaction time for wrong question
CR	Performance Of the task

**TABLE 4:** Language Function

Task modules	Language Function (Corresponding to CCAT)
Find It	Description, Matching, Repetition,
	Reading Comprehension
Match It	Description, Matching, Repetition
Listen It	Description, Matching, Repetition,
	Auditory Comprehension
Say It	Description, Matching, Reading Comprehension
	Reading Comprehension, Expression, Repetition

## IV. Results

The clinical trial involved 27 participants, with 11 assigned to the experimental group receiving module-based therapy. Some participants had mild cognitive impairments in addition to aphasia, therefore, the Say It module, which requires higher cognitive and arithmetic skills, was excluded from this phase. The remaining modules, Find It, Match It, and Listen It, were administered and generally well-received due to their intuitive and accessible design. Results are presented from two perspectives: First, rehabilitation outcomes based on CCAT score changes, and second, insights from ML-based analysis of behavioral performance features.

### A. Rehabilitation Performance

Compares clinical and demographic variables between groups, confirming no significant baseline differences. This supports the validity of subsequent outcome comparisons. Pre and post-intervention CCAT scores, provided by Taipei Veterans General Hospital, were used as the primary metric for assessing language recovery.

#### 1) Intergroup Comparisons

Following the four week intervention 12 sessions, we compared language outcomes between groups. Table 5, presents pre and post intervention CCAT scores for all nine subtests, including means and standard deviations. Paired t-tests assessed within group gains, while intergroup differences evaluated treatment effectiveness. The experimental group showed significant improvement in seven of nine subtests especially in auditory comprehension and written expression indicating that the module based intervention outperformed standard therapy.

**TABLE 5:** Mean Group Data (SD) of CCAT Scores Obtained Pre and Post Therapy

CCAT Score	Control Group (n=16)		Experiment Group (n=11)		P-Value
	Pre	Post	Pre	Post	
Conservation	5.98 (3.44)	6.03 (3.41)	7.13 (2.38)	7.69 (2.55)	0.04*
Description	4.8 (3.22)	5.06 (3.04)	6.07 (2.28)	6.9 (2.5)	0.007**
Matching	10.81 (2.78)	10.88 (2.74)	9.19 (2.73)	10.22 (2.1)	0.004**
Auditory Comprehension	6.83 (2.94)	6.73 (2.92)	7.22 (2.35)	8.2 (2.12)	0.0011**
Expression	5.58 (3.32)	5.5 (3.3)	6.83 (2.43)	7.39 (2.24)	0.028*
Reading Comprehension	6.49 (3.22)	6.41 (3.26)	8.33 (2.91)	8.82 (2.45)	0.167
Repetition	6.23 (3.76)	6.33 (3.73)	8.89 (1.81)	9.56 (1.49)	0.017*
Imitation Writing	8.57 (3.41)	8.34 (3.52)	9.34 (2.68)	9.98 (1.97)	0.00198**
Spontaneous Writing	5.41 (2.86)	5.23 (2.68)	6.68 (2.68)	7.27 (2.62)	0.041*
Total Score	60.71 (25.17)	60.51 (24.68)	69.68 (14.08)	76.02 (14.01)	0.00008***

^*^ Indicates Experimental group showed significant improvement compared to the control group at P < 0.05,

^**^ P<0.01,

^***^ P<0.001 (Paired T-Test).

#### 2) Intragroup Comparisons

Table 6, shows pre and post intervention scores for the control group, with no significant gains across any subtests indicating limited impact from conventional therapy during the study period. In contrast, Table 7, presents results for the experimental group, revealing significant improvements in seven of nine subtests. These gains span both receptive and expressive domains, highlighting the effectiveness of the interactive modules in driving meaningful rehabilitation progress.

**TABLE 6:** Mean Group Data (SD) of CCAT Scores Obtained Pre and Post Traditional Therapy

CCAT SCORE	Control Group (n=16)		P-Value
	Pre	Post	
Conservation	5.98 (3.44)	6.03 (3.41)	0.71
Description	4.8 (3.22)	5.06 (3.04)	0.26
Matching	10.81 (2.78)	10.88 (2.74)	0.55
Auditory Comprehension	6.83 (2.94)	6.73 (2.92)	0.49
Expression	5.58 (3.32)	5.5 (3.3)	0.56
Reading Comprehension	6.49 (3.22)	6.41 (3.26)	0.74
Repetition	6.23 (3.76)	6.33 (3.73)	0.52
Imitation Writing	8.57 (3.41)	8.34 (3.52)	0.23
Spontaneous Writing	5.41 (2.86)	5.23 (2.68)	0.3
Total Score	60.71 (25.17)	60.51 (24.68)	0.74

Indicates significant improvement observed within the control group at P<0.05,**P<0.01,***P<0.001(Paired T-Test)

**TABLE 7:** Mean Group Data (SD) of CCAT Scores Obtained Pre- and Post-Module Therapy

CCAT Score	Experiment Group (n=11)		
	Pre	Post	P-Value
Conservation	7.13 (2.38)	7.69 (2.55)	0.02*
Description	6.07 (2.28)	6.9 (2.5)	0.0004***
Matching	9.19 (2.73)	10.22 (2.1)	0.00085***
Auditory Comprehension	7.22 (2.35)	8.2 (2.12)	0.000875***
Expression	6.83 (2.43)	7.39 (2.24)	0.069
Reading Comprehension	8.33 (2.91)	8.82 (2.45)	0.102
Repetition	8.89 (1.81)	9.56 (1.49)	0.0137*
Imitation Writing	9.34 (2.68)	9.98 (1.97)	0.0085**
Spontaneous Writing	6.68 (2.68)	7.27 (2.62)	0.0228*
Total Score	69.68 (14.08)	76.02 (14.01)	2.26E-05***

Indicates significant improvement observed within the control group at P<0.05,**P<0.01,***P<0.001(Paired T-Test)

#### 3) Case Wise Improvements

**FIGURE 6.**
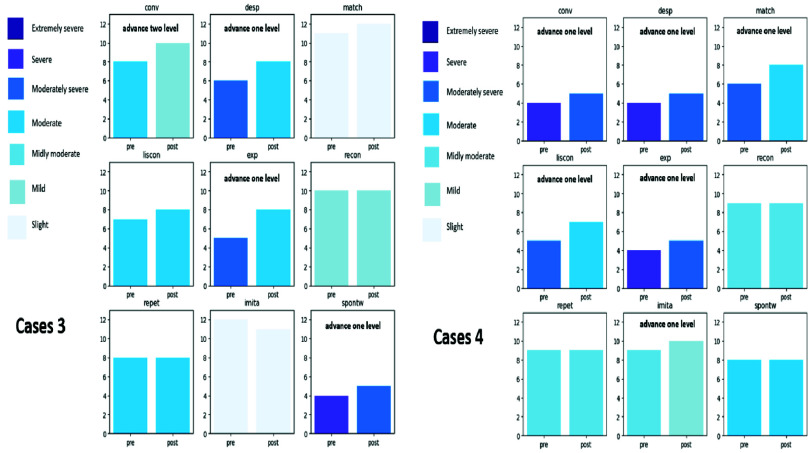
Representative patients demonstrating significant language gains (Cases 3 and 4).

Several experimental group participants showed substantial individual gains. Fig. 5, shows enhanced performance in conversation, semantic matching, and reading comprehension. Fig. 6, illustrates improvements across six metrics, reinforcing the personalized benefit of the gamified therapy modules.

### B. Machine Learning

Two classification pipelines were evaluated, one using CCAT subtest scores, and another using behavioral data (GameData) from the therapy modules. Table 8, summarizes the feature statistics across modules. LR achieved 91.7% accuracy with CCAT input, with the Expression subtest as the top predictor. For GameData, k-NN performed best, with 74.1% accuracy in both Find It and Listen It modules. Key predictors included CorrectRate, MeanWrongTime, and MeanReactTime.

**TABLE 8:** Number of Task Data and Feature Statistics

Modules	D:A	Corresponding Feature	MEan(SD)
Find It		ReactTime CorrectReactTime WrongReactTime CorrectRate	16.41(8.914) 17.12(9.514) 8.2(8.389) 0.829(0.174)
Match It	3:8	ReactTime CorrectReactTime WrongReactTime CorrectRate	18.218(17.223) 17.695(18.107) 18.671(15.235) 0.727(0.236)
Listen It		ReactTime CorrectReactTime WrongReactTime CorrectRate	27.631(13.046) 23.638(13.398) 15.45(18.948) 0.796(0.291)

#### 1) CCAT as a Feature

**FIGURE 7.**
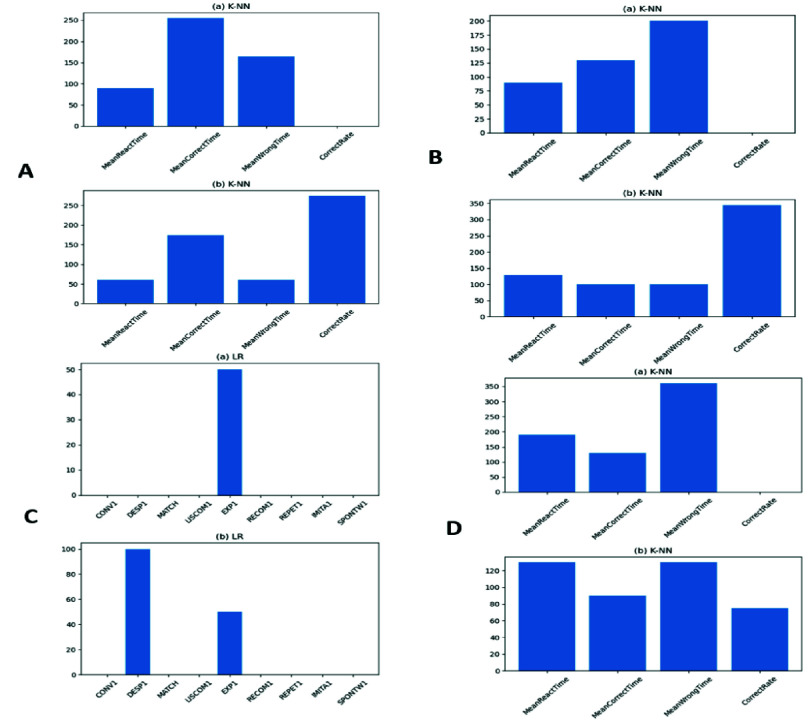
Permutation importance results across four therapy modules. (A) Listen It, (B) Find It, (C) CCAT subtests, and (D) Match It. Each plot shows the relative importance of key features used by classifiers to estimate aphasia severity or predict recovery trends.

Table 9, presents classification results using CCAT subtest scores as input features. Overall, model performance remained consistent between raw and standardized datasets, with only minor variation. Among the classifiers tested, SVM, RF, and LR demonstrated strong predictive accuracy, with LR achieving the highest at 91.7%. To enhance interpretability, feature importance analysis was applied. In the raw data condition, the LR model identified the Expression subtest as the most influential predictor Fig. 7. These results underscore the diagnostic utility of expressive and descriptive language functions in distinguishing aphasia from dysarthria.

**TABLE 9:** CCAT Results of the Five Algorithms

Accuracy	K-NN	SVM	RF	DT	LR
Origin	0.769	0.917	0.917	0.898	0.917
Standardization	0.796	0.917	0.917	0.898	0.917

#### 2) GameData as Feature

Find It Module Behavioral features from the Find It module yielded the highest classification accuracy using the k-NN algorithm on both raw and standardized datasets, reaching 74.1% accuracy Table 10. In the raw feature setting, MeanWrongTime was the most influential predictor, followed by MeanReactTime and MeanCorrectTime Fig. 7. These results suggest that both response accuracy and reaction patterns are key indicators in semantic comprehension classification. Match It Module For the k-NN algorithm again delivered the best classification performance, with accuracies of 66.7% (raw) and 67.6% (standardized) datasets Table 11. In the raw feature setting, MeanWrongTime was the most important feature, followed by MeanReactTime and MeanCorrectTime Fig. 7. These findings indicate that both response latency and error timing are informative for identifying impairments in associative semantic tasks. However, the overall accuracy of the GameData based classifiers remains lower than CCAT based models, primarily because behavioral data are more heterogeneous and context dependent. Unlike structured CCAT tasks, in game responses vary with attention, learning rate, and user engagement. Future iterations will explore temporal sequence analysis, richer semantic context embeddings, and multimodal fusion (audio, gaze, facial cues) to strengthen predictive robustness and clinical equivalency.

**TABLE 10:** Find it Results of the Five Algorithms

Accuracy	K-NN	SVM	RF	DT	LR
Origin	0.741	0.528	0.685	0.611	0.565
Standardization	0.741	0.713	0.685	0.611	0.713

**TABLE 11:** Match it Results of the Five Algorithms

Accuracy	K-NN	SVM	RF	DT	LR
Origin	0.667	0.50	0.537	0.565	0.407
Standardization	0.676	0.48	0.537	0.565	0.472

For the Listen It module, the k-NN algorithm again achieved the highest accuracy on both raw and standardized datasets, each reaching 74.1% Table 12. In the raw feature analysis, MeanCorrectTime was the most influential, followed by MeanReactTime and MeanWrongTime Fig. 7. These results highlight the importance of response timing and accuracy in auditory comprehension tasks.

**TABLE 12:** Listen it Results of the Five Algorithms

Accuracy	K-NN	SVM	RF	DT	LR
Origin	0.741	0.704	0.704	0.648	0.667
Standardization	0.741	0.676	0.704	0.648	0.704

### C. Model Interpretation and Clinical Insights

Across classification tasks, both k-NN and SVM performed well, especially with data from aphasia related modules. These modules generate clear time and accuracy features that effectively distinguish aphasia from dysarthria. The superior performance of k-NN is likely due to its reliance on distance based clustering, which handles small dataset variations well. In contrast, SVM showed greater sensitivity to subtle fluctuations, potentially affecting its robustness. When using CCAT subtest scores, Description and Expression emerged as the most influential features, consistent with clinical understanding, while dysarthria impacts articulation, aphasia disrupts broader language formulation. Tasks requiring grammatically structured responses from abstract prompts are particularly challenging for aphasia patients. Feature importance analysis showed raw data emphasized timing metrics, whereas standardized data shifted focus toward CorrectRate, a more stable and interpretable measure. Unlike timing features, CorrectRate normalizes performance and is less prone to outliers. These findings are supported by empirical results and clinician feedback, though further validation is needed for broader generalization. In practice, the explainability outputs are presented through a clinician facing visualization dashboard. Each model decision is accompanied by bar chart overlays showing the top contributing features (CorrectRate, MeanReactTime, MeanWrongTime) together with predicted severity and confidence levels. Clinicians at Taipei Veterans General Hospital reported that these visual summaries help them verify whether model reasoning aligns with observed patient behavior. For instance, cases with high MeanWrongTime but stable comprehension patterns often indicate attention or motor coordination deficits rather than linguistic impairment, prompting therapists to adjust training focus. Future iterations will incorporate structured clinician feedback and case based interpretability summaries to further enhance transparency and support explainable decision making in daily rehabilitation practice.

### D. Correlation Between CCAT and GameData

**FIGURE 8.**
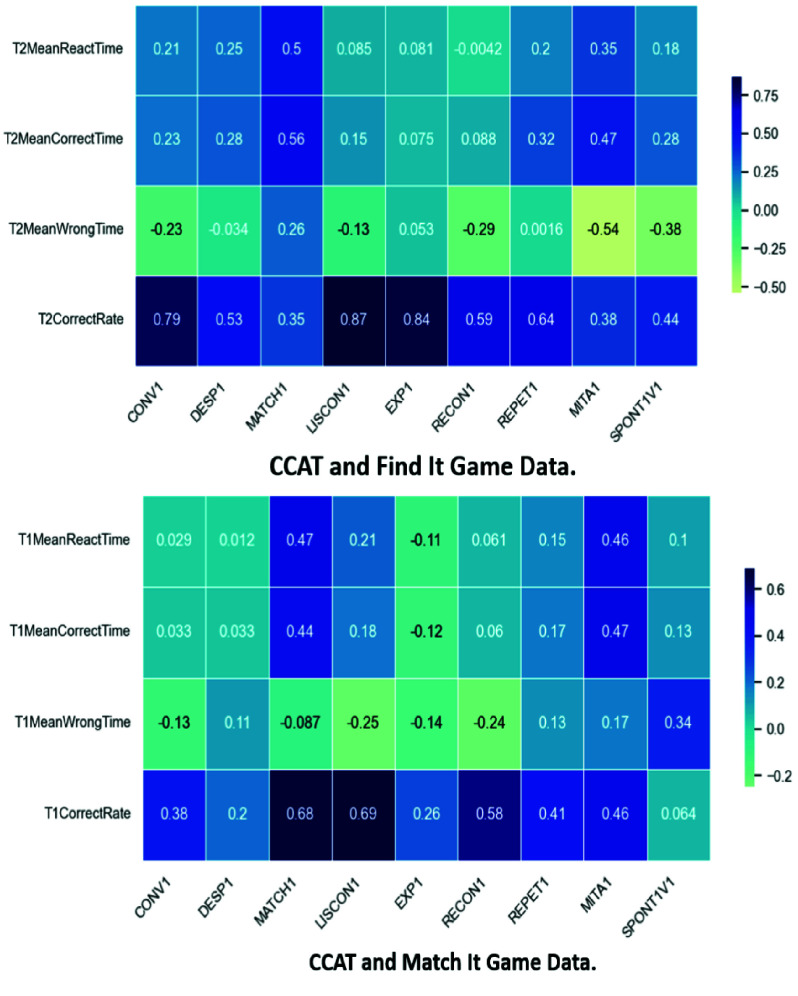
Correlation between CCAT and GameData from find it and match it modules.

**FIGURE 9.**
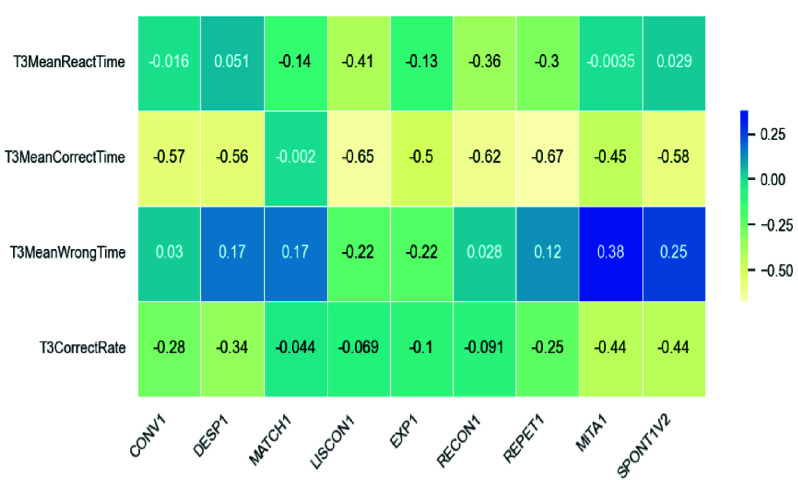
Correlation between CCAT and listen it GameData.

We performed Pearson correlation analysis between pre test CCAT scores and in game GameData features from the experimental group to evaluate alignment between clinical outcomes and module performance. As shown in Fig. 8, shows stronger correlations for the Match It module, particularly in CorrectRate and MeanCorrectTime, with Matching, Reading, and Picture Description subtests. In contrast, Fig. 9, indicates weak or no significant correlations for Listen It, suggesting its current design does not yet align well with clinical measures. These results highlight CorrectRate as a promising proxy for assessment in well-aligned modules and underscore the need to refine modules like Listen It for better clinical relevance.

## V. Discussion

Our clinical findings show that the experimental group significantly outperformed the control group in CCAT assessments after a four week intervention ( $p < 0.001$). Notable gains were observed in item matching, auditory comprehension, and descriptive language tasks ( $p < 0.01$). The control group showed no significant improvements, highlighting the limited short-term efficacy of conventional therapist led therapy. The proposed module-based system enabled effective, engaging gamified rehabilitation. Due to the prevalence of mild cognitive impairment, the Listen It module was adapted for arithmetic based tasks. The more cognitively demanding Say It module was excluded for consistency and will be assessed in future studies. In the machine learning analysis, repeated 10-fold cross-validation was used to reduce overfitting risks. Logistic Regression achieved 97.1% accuracy using CCAT subtest scores, while k-NN performed best using in game features, with accuracies of 74.1%, 67.6%, and 74.1% across modules. The observed performance gap between CCAT based and GameData based classifiers highlights the challenge of modeling spontaneous, ecologically valid behaviors. In game data capture real world variability motor delays, adaptive learning, fatigue, or motivational factors that standardized tests minimize. While this realism introduces noise, it also reflects true functional communication. To bridge this gap and achieve clinical equivalency, future work will implement temporal sequence modeling of response trajectories, contrastive and self supervised learning to enhance feature stability, and multimodal integration of speech, facial, and gaze cues. These refinements are expected to improve autonomous severity estimation and align ML based outputs with therapist rated outcomes. These results underscore the novel integration of gamified modules with real time, explainable ML assessment, providing clinically interpretable feedback that supports treatment personalization and bed-to-home continuity of care.

The system has undergone hospital based pilot validation in collaboration with certified clinicians at Taipei Veterans General Hospital. All patient data collection and intervention procedures were conducted under Institutional Review Board (IRB) approval, in accordance with the Declaration of Helsinki and hospital ethical guidelines. The Language Interactive Lab is implemented as a SaMD prototype developed in compliance with TFDA requirements, IEC 62304 software life cycle standards, and ISO 14971 risk management guidelines. The prototype currently operates under hospital investigational use authorization and is being prepared for formal TFDA pre market submission. These regulatory grade frameworks ensure traceability, cybersecurity, and patient data integrity, supporting the system’s transition from a research platform to a clinically deployable digital therapeutic. Future work will extend to multi site clinical trials and full regulatory approval to enable seamless integration with electronic health record (EHR) systems and home based rehabilitation programs. The statistically significant improvements achieved across multiple CCAT subtests provide direct evidence of real world therapeutic effectiveness. Because the system operates on standard personal computers and supports remote connectivity, it offers a practical and affordable solution for extending aphasia rehabilitation to underserved or rural regions where access to specialized speech language therapy is limited. While the present study provides statistically significant results, the relatively small cohort size (n= 27) limits broad generalizability. Future multi center trials with larger, more diverse populations will be essential to validate robustness across clinical contexts.

## VI. Conclusion and Future Work

This study confirms the feasibility and clinical effectiveness of an interactive, gamified rehabilitation platform that couples real time performance capture with explainable machine learning assessment. The approach improves language outcomes and shows strong alignment between in game behavioral metrics and recovery trajectories, supporting clinically interpretable, personalized neurorehabilitation. The system offers potential for scalable, cost effective deployment in clinical and home settings, particularly where therapist access is limited. Future work will integrate virtual reality to simulate real world communication and enhance engagement. We also plan to add wearable sensors (Electroencephalography (EEG), EMG (Electromyography), eye tracking, IMUs (Inertial Measurement Units), heart rate) for multimodal data capture. This fusion of AI, VR, and biosensing will enable personalized therapy, improve diagnostic precision, and support an adaptive rehabilitation ecosystem.

While these directions represent exciting opportunities for future clinical translation, the current study also has several practical limitations that inform our next steps. Despite these promising outcomes, several limitations should be acknowledged. First, the Say It module, which involves complex lexical retrieval and verbal expression, was excluded from the current trial to ensure uniform task difficulty across participants and to reduce cognitive load for individuals with mild cognitive impairment. Future iterations will reintegrate this module using adaptive difficulty scaling and speech driven reinforcement to evaluate expressive language recovery. Second, variability in cognitive comorbidities such as attention and motor coordination may have influenced in game behavioral measures, contributing to performance variance. Ongoing development focuses on multimodal feature fusion combining speech, facial, and physiological cues to better disentangle linguistic from cognitive or motor factors. Finally, the current four week pilot study represents a short term intervention window. Planned longitudinal trials will extend to 12–24 weeks, enabling long term monitoring of functional recovery, generalization effects, and sustained engagement in home based rehabilitation contexts.
